# Loss of attachment promotes proline accumulation and excretion in cancer cells

**DOI:** 10.1126/sciadv.adh2023

**Published:** 2023-09-06

**Authors:** Steven E. Pilley, Marc Hennequart, Anke Vandekeere, Julianna Blagih, Nathalie M. Legrave, Sarah-Maria Fendt, Karen H. Vousden, Christiaan F. Labuschagne

**Affiliations:** ^1^ The Francis Crick Institute, 1 Midland Road, London NW1 1AT, UK.; ^2^ Laboratory of Cellular Metabolism and Metabolic Regulation, VIB-KU Leuven Center for Cancer Biology, VIB, Leuven, Belgium.; ^3^ University of Montreal, Maisonneuve-Rosemont Hospital Research Centre, 5414 Assomption Blvd, Montreal H1T 2M4, Canada.; ^4^ Human Metabolomics, Faculty of Natural and Agricultural Sciences, North-West University (Potchefstroom Campus), 11 Hoffman Street, Potchefstroom 2531, South Africa.

## Abstract

Previous studies have revealed a role for proline metabolism in supporting cancer development and metastasis. In this study, we show that many cancer cells respond to loss of attachment by accumulating and secreting proline. Detached cells display reduced proliferation accompanied by a general decrease in overall protein production and de novo amino acid synthesis compared to attached cells. However, proline synthesis was maintained under detached conditions. Furthermore, while overall proline incorporation into proteins was lower in detached cells compared to other amino acids, there was an increased production of the proline-rich protein collagen. The increased excretion of proline from detached cells was also shown to be used by macrophages, an abundant and important component of the tumor microenvironment. Our study suggests that detachment induced accumulation and secretion of proline may contribute to tumor progression by supporting increased production of extracellular matrix and providing proline to surrounding stromal cells.

## Teaser

Despite lower general amino acid synthesis, detached cancer cells maintain proline synthesis, accumulation, and excretion.

## INTRODUCTION

Oncogenic transformation is associated with many metabolic changes, including alterations in metabolic pathways that support energy production, the production of macromolecules to allow cell growth, and the adaptation of systems to allow for redox control. Complex metabolic rewiring in cancer cells contributes to their ability to proliferate and survive in nutrient-poor conditions and abnormal environments. Loss of matrix attachment, as encountered during invasion and metastasis, has been shown to lead to increased oxidative stress, requiring a compensatory increase in antioxidant capacity to allow cell survival (*1*, *2*). Previous studies have identified several metabolic responses that contribute to the ability of cells to survive detachment, including increased reductive carboxylation and mitophagy, that help to limit reactive oxygen species (ROS). The switch to reductive glutamine metabolism also generates oxidised nicotinamide adenine dinucleotide (NAD^+^) through the production of malate by malate dehydrogenase and lactate by lactate dehydrogenase A (*1*), helping to maintain redox balance in cells where mitochondrial respiration is limited. While antioxidant capacity is important in allowing cancer cells to survive during dissemination in the blood or lymph (*3*), the availability of oxidized NAD^+^ can also be a limiting factor for pathways such as serine synthesis (*4*). Furthermore, many cancer cells limit respiration even under conditions of adequate oxygen when increased requirements for NAD^+^ to support the many metabolic reactions needed for tumor growth exceeds the demand for adenosine triphosphate (ATP) (*5*).

In addition to cell intrinsic metabolic changes that promote successful dissemination of cancer cells, factors within the primary tumor microenvironment (TME) also play an important role in determining metastatic potential (*6*–*8*). Cells of the TME provide several types of support to cancers, including metabolic interactions that can help provide nutrients and shape a protumorigenic milieu (*9*). In addition to the cells of the TME, the extracellular matrix also plays an important role in determining invasive capacity, with changes in collagen contributing to both the promotion and inhibition of metastasis (*10*). While much of the collagen in the TME is produced by surrounding fibroblasts, cancer cells also produce collagen, which has been shown in pancreatic cancers to be strongly tumor promoting (*11*).

Proliferating cancer cells have a high demand for amino acids, and while uptake of exogenous amino acids has been shown to be the major contributors to cell mass (*12*), pathways of de novo synthesis of nonessential amino acids can play an especially important role in nutrient-poor conditions. For example, increased activity of the serine synthesis pathway has been shown to contribute to several types of cancer, and targeting de novo serine synthesis pathways can effectively reduce tumor growth, particularly in environments of limited exogenous serine (*13*, *14*). Proline is also important to support cancer progression, with evidence that de novo synthesis of proline is modulated in response to proline availability (*15*, *16*). Increased expression of the proline synthesis pathway enzyme pyrroline-5-carboxylate reductase 1 (PYCR1) has been noted in several tumor types and is a marker of poor prognosis (*15*, *17*). Unlike serine synthesis, which consumes NAD^+^, mitochondrial proline synthesis regenerates NAD^+^. This activity plays an important role under conditions of redox disturbance, such as hypoxia (*18*), *IDH1* mutation (*19*) and in response to transforming growth factor–β stimulation (*20*). On the other hand, mitochondrial proline catabolism through proline dehydrogenase (PRODH) supplies electrons to the ubiquinone pool to support oxidative phosphorylation, providing ATP to help cell survival under conditions of starvation (*21*). This activity is also important for the survival of detached cells, where proline availability enhances ATP generation via PRODH-dependent proline degradation (*22*, *23*). HRAS transformed breast cancer cells show increased uptake of proline when grown as spheroids and enhanced PRODH-mediated proline catabolism is required for three-dimensional (3D) growth and metastasis but not primary tumor growth (*23*). However, PRODH also drives increased ROS and cell death and has been suggested to function as a mediator of p53-driven tumor suppression (*24*–*26*). In addition to redox control, proline also directly contributes to protein synthesis, where it plays an essential structural role (*27*). Collagen contains a high proportion of proline residues, and limiting proline production has been shown to impede collagen deposition and limit cancer progression (*28*). The ability of detached tumor cells to produce proline may therefore contribute to tumor progression, particularly in a nutrient- and oxygen-poor environment.

Here, we show that detachment of cancer cells from their matrix leads to increased proline accumulation and secretion. This effect results from a selective retention of proline synthesis, despite a reduction in the synthesis of other amino acids that correlates with slower proliferation, and lower incorporation of proline into proteins. However, notwithstanding this general reduction in the use of proline for protein production, detached cancer cells increase synthesis of proline-rich collagen. We also show that the excess proline can be taken up by other cancer-associated cells.

## RESULTS

**Fig. 1.**
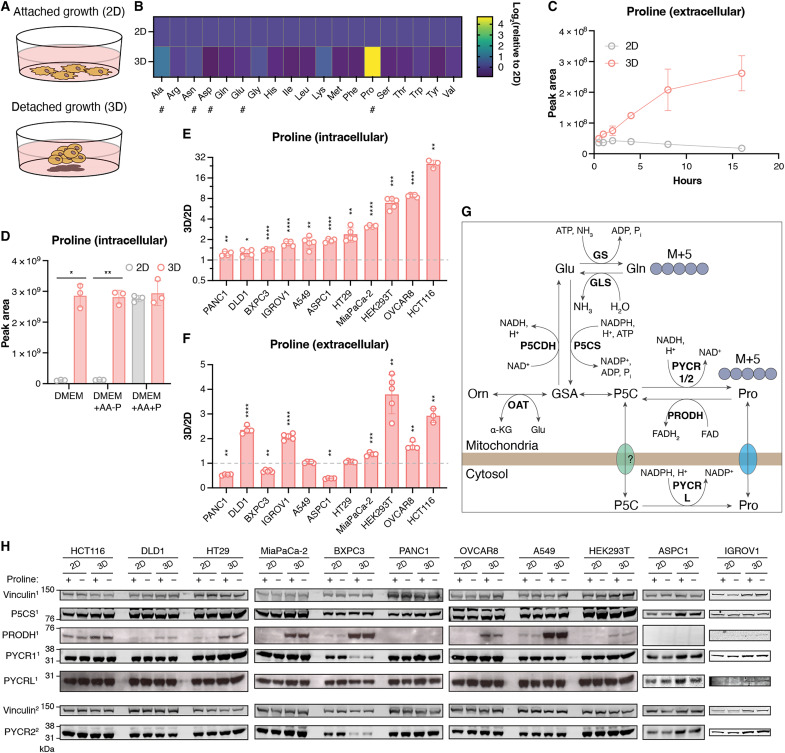
3D cancer cells accumulate and excrete proline. (**A**) Schematic showing 2D and 3D growth. (**B**) Heatmap of the log 2 fold change of the level of each measured intracellular amino acid in 3D to 2D. “#” indicates amino acid is not present in DMEM. (**C**) Level of proline detected in the media of 2D and 3D cells at defined time points after media replenishment. Performed in DMEM. (**D**) Intracellular proline levels in 2D and 3D cells incubated in the indicated media normalized to cell number. (**E**) Ratio of total intracellular proline in 3D culture to 2D culture for each cell line. Performed in DMEM+AA-P. (**F**) Ratio of total extracellular proline in 3D culture to 2D culture for each cell line. Performed in DMEM+AA-P. (**G**) Schematic of proline metabolism. (**H**) Western blot showing expression of the enzymes involved in proline metabolism of various cell lines in 2D and 3D in DMEM+AA+P or DMEM+AA-P. All metabolites measured using LC-MS. All data presented as means ± SD, *n* ≥ 3 technical replicates. [(B) to (D)] Representative of at least two experiments. (D) Brown-Forsythe and Welch analysis of variance (ANOVA) corrected for multiple comparisons and [(E) and (F)] multiple Welch unpaired *t* tests: *P < 0.05, **P < 0.01, ***P < 0.001, ****P < 0.0001.

Switching normally adherent cancer cells (attached 2D culture) to nonadherent growth conditions (detached 3D culture) provides a very simplified model of the loss of stromal support that occurs during metastasis (Fig. 1A) and has provided some important information about the metabolic response of cancer cells to this process (*1*, *2*, *23*). To examine how detachment affects amino acid metabolism, we monitored changes in intracellular amino acid levels in HCT116 (a colorectal cancer cell line) following detachment. While the levels of most amino acids did not show a clear change, there was a strong increase in proline levels in the detached cells (Fig. 1B) compared to that detected in attached cells. Detached HCT116 cells also excreted proline, which accumulated in the extracellular medium over time (Fig. 1C). Notably, these experiments were carried out in Dulbecco’s modified Eagle’s medium (DMEM), a tissue culture medium lacking alanine, aspartate, asparagine, glutamate, and proline. To assess whether the lack of other amino acids in the tissue culture medium was affecting proline accumulation, we adapted DMEM to contain all the other amino acids, adding or removing only proline (DMEM+AA+P and DMEM+AA-P, respectively; Fig. 1D). Again, in the absence of exogenous proline, HCT116 cells grown in 3D accumulated high levels of proline compared to cells grown in 2D. Adding exogenous proline led to an increase in intracellular proline in 2D cells to match those seen in 3D cells, where the proline levels were not altered by the addition of extracellular proline. In these experiments, metabolite extracts were normalized to cell number, and to ensure that the observed difference in intracellular proline levels was not due to differences in cell volume in 2D and 3D, we repeated the experiment normalized to protein content. Once again, proline accumulated to much higher levels in 3D cells than in 2D cells (fig. S1A). These results suggest that at a certain intracellular concentration, cells excrete proline, and that 3D cells exceed this level and so excrete the excess. All subsequent studies were carried out in medium containing all amino acids or lacking only proline.

In contrast to the present results, previously published data showed that when transformed breast cells (MCF10A-HRAS^V12^) are cultured in soft agar—which also limits attachment—in medium containing proline, the steady-state levels of intracellular proline are lower in 3D than in 2D (*23*). To understand this difference, we repeated the experiments with MCF10A-HRAS^V12^ cells and found that proline levels in detached cells grown in the absence of proline were higher than in attached cells, consistent with the results from other cell lines (fig. S1B). Independent experiments using MCF10A-HRAS^V12^ cells grown in soft agar confirmed the published decrease in proline levels in these cells when grown in the previously described conditions (in medium containing proline) (fig. S1C). These results suggest that proline metabolism changes in different culture conditions.

To determine whether the accumulation of proline is a general response of cancer cells to detachment, we examined a series of different cancer cell lines (Fig. 1E). While proline levels were highest in HCT116 cells, many of the other cell lines showed twice or more intracellular proline in detached compared to attached cells (Fig. 1E). Similarly, many of other cancer cell lines also excreted more proline when grown in 3D compared to 2D (Fig. 1F).

In cells, proline is synthesized from glutamate-5-semialdehyde (GSA) and pyrroline-5-carboxylate (P5C), which can be derived from glutamate or ornithine, through reactions catalyzed by mitochondrial enzymes PYCR1 and PYCR2 (Fig. 1G). The oxidation of proline back to P5C by PRODH occurs at the mitochondrial inner membrane, providing electrons to the mitochondrial electron transport chain to support ATP and ROS production. Pyrroline-5-carboxylate reductase 3 (PYCRL) in the cytosol also produces proline from P5C; the PYCRL and P5C synthase (P5CS, encoded by *ALDH18A1*) catalyzed reactions both use NADPH (*29*). Proline cycling through these cytosolic and mitochondrial reactions could transfer reducing equivalents into the mitochondria. In multiple cell lines, the expression of most of the proline metabolism enzymes was not altered in 3D compared to 2D, in the presence or absence of proline (Fig. 1H). Exceptionally, PYCR1 and PYCR2 expression was decreased in detached BXPC3 cells. By contrast, an increase in PRODH expression was seen in most detached cells compared to cells grown in 2D, although this was not evident in PANC1 or ASPC1 cells, where PRODH levels were not detectable. While increased PRODH activity could contribute to the increased ROS seen in detached cells (*1*, *2*), it is unlikely to explain the accumulation and secretion of proline in response to detachment. PRODH levels are the same in 2D cells cultured with or without proline, showing that PRODH up-regulation is not a response to increased proline availability.

**Fig. 2.**
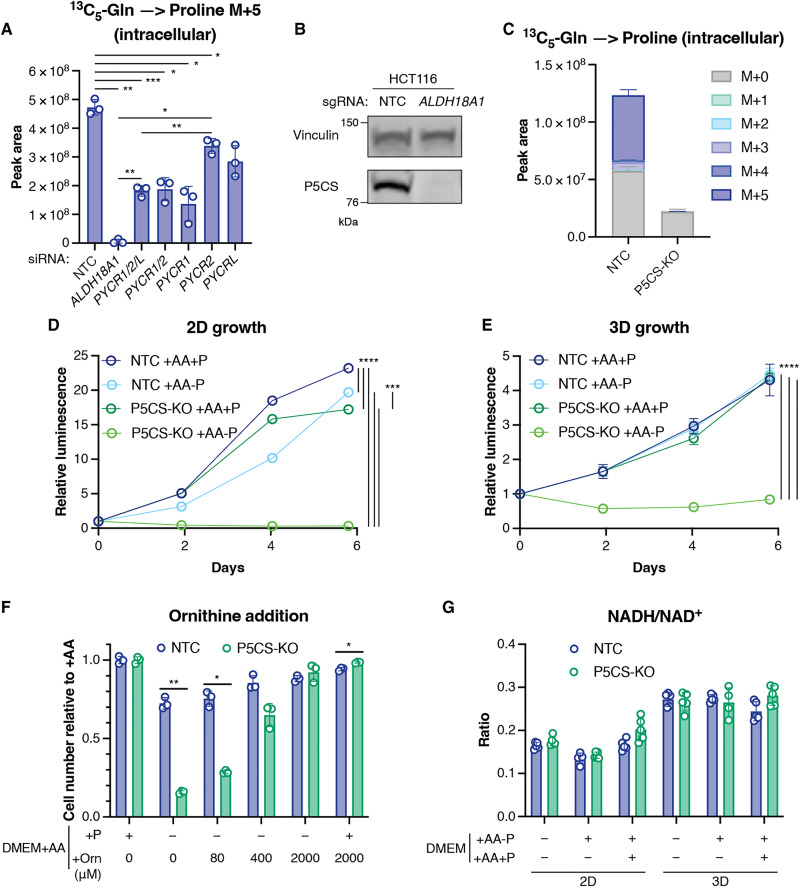
Proline synthesis in 3D cultures is not required to maintain proliferation. (**A**) Intracellular proline M+5 in HCT116 cells incubated in DMEM+AA+P with ^13^C_5_-glutamine transfected with the siRNAs targeting the genes indicated. *ALDH18A1* encodes P5CS. (**B**) Western blot showing P5CS expression in HCT116 NTC and P5CS-KO cell lines. Vinculin is the loading control. (**C**) Intracellular proline in HCT116 NTC and P5CS-KO cells cultured in DMEM+AA-P with ^13^C_5_-glutamine. Growth of HCT116 NTC and P5CS-KO cells in (**D**) 2D and (**E**) 3D culture in DMEM+AA+P or DMEM+AA-P with or without proline, measured using CellTiter-Glo. (**F**) Cell number of HCT116 NTC and P5CS-KO cells cultured in DMEM+AA+P or DMEM+AA-P and where indicated, increasing concentrations of ornithine (Orn; 80, 400 and 2000 μM). Normalized to DMEM+AA+P. (**G**) Ratio of intracellular NADH to NAD^+^ in HCT116 NTC and P5CS-KO cells in 2D and 3D culture in the indicated media. All metabolites measured using liquid chromatography–mass spectrometry (LC-MS). All data are presented as means ± SD, *n* = 3 technical replicates except for (D) and (E) *n* = 6. All data are representative of at least two experiments. (A) Brown-Forsythe and Welch ANOVA, [(D) and (E)] two-way ANOVA, and [(F) and (G)] multiple unpaired Welch *t* tests. All corrected for multiple comparisons. *P < 0.05, **P < 0.01, ***P < 0.001, ****P < 0.0001.

Previous work has shown that in hypoxic conditions, increased activity of PYCR1 is necessary to maintain NAD^+^ to support tricarboxylic acid cycle activity and proliferation—a response that also increases proline production (*18*). Redox stress has also been noted in cells following detachment (*1*, *2*), suggesting that the accumulation of proline seen in these cells could be a by-product of up-regulation of reactions that generate NAD^+^. To directly assess the effect of inhibition of de novo proline synthesis on maintenance of proliferation in detached cells, we used small interfering RNA (siRNA) to deplete various enzymes in the pathway (Fig. 2A). Depletion of the PYCR enzymes did not completely inhibit proline production in this system, most likely reflecting redundance of function and an incomplete depletion of all three enzymes. However, P5CS depletion efficiently blocked the generation of labeled proline from labeled glutamine. Using CRISPR to generate a control cell line (NTC) and an HCT116 cell line lacking P5CS (P5CS-KO), we confirmed that these cells were unable to make proline from glutamine (Fig. 2, B and C). As expected, this defect resulted in a failure of P5CS-KO cells to proliferate in the absence of extracellular proline both in 2D and 3D (Fig. 2, D and E). Proline can also be synthesized from ornithine, via the action of ornithine aminotransferase. While the tissue culture media used for these studies does not contain ornithine, the growth of HCT116 P5CS-KO cells in the absence of exogenous proline can be rescued by providing ornithine (Fig. 2F). These results show that although ornithine can support proline synthesis in these cells, de novo ornithine synthesis is not sufficient to support the proline requirements of these cells. Furthermore, complete rescue of P5CS-KO growth in the absence of proline could only be achieved using supraphysiological levels of ornithine, as the level of ornithine found in human circulation (~80 μM) only partly rescued proliferation. Loss of P5CS did not affect the proliferation of HCT116 cells provided with extracellular proline in 2D or 3D (Fig. 2, D and E), demonstrating that the synthesis of proline and oxidation of NADH (reduced form of NAD^+^) is not a requirement for the growth of detached cells. Furthermore, while an increased NADH/NAD^+^ ratio (indicative of a response to a redox imbalance) was detected in detached cells, this was not exacerbated by loss of the ability to carry out proline synthesis (Fig. 2G). These results indicate that the increased proline level seen in detached cells is not due to an increased dependence on proline synthesis to redress a redox imbalance.

**Fig. 3.**
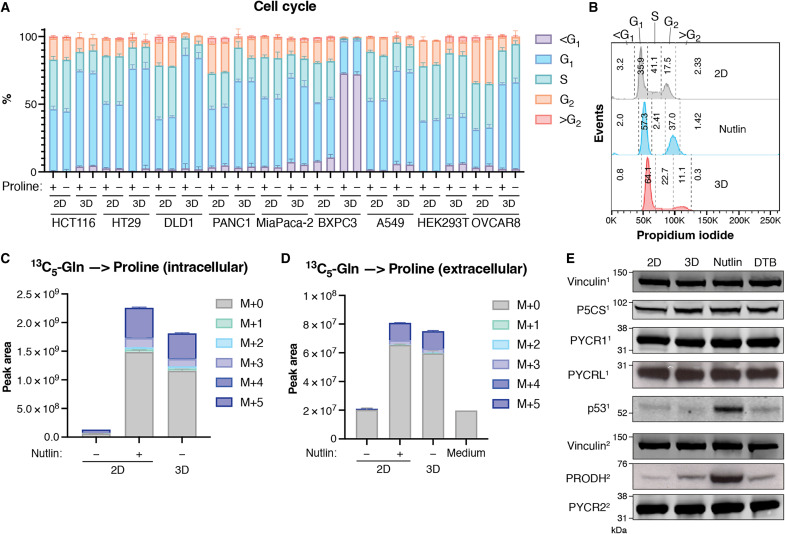
Proline accumulation correlates with G_1_ accumulation in 3D culture. (**A**) Quantification of cell cycle analysis on a panel of cell lines grown in 2D or 3D in DMEM+AA+P or DMEM+AA-P. (**B**) Representative histograms of cells cultured in 2D with or without 10 μM Nutlin or in 3D in DMEM+AA-P stained with propidium iodide to show cell cycle distribution. Numbers indicate the percentage of cells in each cell cycle stage. (**C**) Intracellular and (**D**) extracellular proline of HCT116 cells cultured in 2D with or without 10 μM Nutlin or in 3D in DMEM+AA-P with ^13^C_5_-glutamine. “Medium” shows proline level in the medium before it was put onto cells. (**E**) Western blot showing the expression of the indicated proteins in HCT116 cells cultured in 2D, in 3D, with 10 μM Nutlin or under a DTB in DMEM+AA-P. Vinculin is the loading control. The numbers after each protein refer to the membrane the antibody was incubated with. All metabolites measured using LC-MS. All data are presented as means ± SD, *n* = 3 technical replicates, except for medium samples where *n* = 1.

To understand the underlying cause of increased proline in detached cells, we considered this could be the result of increased proline synthesis or decreased proline utilization. Proline can be used in various metabolic pathways to produce glutamate and ornithine, but we were unable to detect any labeling of these metabolites from labeled proline in cells grown either in 2D or 3D, suggesting that a decreased use of proline in these pathways is not the reason for the accumulation of proline in 3D cells. We noted that cells in 3D grew more slowly than cells in 2D (Fig. 2, D and E), with an increased proportion of cells in G_1_ in all cell lines except human embryonic kidney (HEK) 293T and BXPC3 [which showed a large accumulation of sub-G_1_ cells indicating extensive cell death in 3D (Fig. 3A) possibly causing the drop in PYCR1 and 2 levels observed in 3D culture (Fig. 1H)]. Nutlin induced p53 activation, and inhibition of G_1_ cell cycle progression (Fig. 3, B and E) led to an accumulation and excretion of proline in attached cells that was similar to that seen following detachment (Fig. 3, C and D). Although there were some differences in the levels of other amino acids between cells with and without Nutlin, by far the largest difference was in proline (fig. S2A). Furthermore, induction of cell cycle arrest using an alternative method, a double thymidine block (DTB), also caused 2D cells to accumulate and excrete proline (fig. S2, B to D). While cells cultured at a lower temperature or in a lower concentration of serum showed similar reductions in proliferation, only reduced serum led to an increase in proline levels in 2D (fig. S2, E to H). Proliferation in reduced serum but not at lower temperature was associated with a delay in G_1_ progression similar to that seen in cells cultured in 3D (fig. S2I).

Nutlin- or DTB-induced increase in proline accumulation and secretion was accompanied by increased expression of PRODH (an established p53 target gene) (*30*) but without changes in expression of the proline synthesis enzymes—again similar to the response seen in detached cells (Fig. 3E). PRODH was also found to increase in 2D cells cultured in 1% serum but not at 33°C (fig. S2J). However, levels of p53 do not increase in HCT116 cells in 3D or under DTB (Fig. 3E), and many of the other cell lines found to accumulate proline in 3D contain mutations in *TP53*, which are known to have lost most wild-type activity, including the ability to inhibit proliferation. Together, the results suggest that delay or inhibition of cell cycle progression—and not simply reduction in growth rate—are associated with proline accumulation in 3D cells and that while p53 activation leads to a robust increase in PRODH, p53 activation is not necessary for PRODH induction or responsible for proline accumulation.

To explore the link between the increase in the levels of PRODH protein and proline accumulation, we first analyzed proline synthesis in HCT116 cells depleted of PRODH (fig. S3A). PRODH suppression caused a slight increase in proline levels in cells cultured in 2D but a decrease in cells cultured in 3D (fig. S3B). However, in agreement with other studies (*31*, *32*) in both 2D and 3D, PRODH suppression led to reduced proliferation and increased cell death, irrespective of the presence of exogenous proline (fig. S3, C and D). To follow the fate of proline in these cells, we used a fully deuterated isotopolog of proline (D_7_-proline). We could not detect any deuterated ornithine or glutamate in cells cultured in 2D or 3D, and the intermediates P5C and GSA were not detected at all. It has been suggested that the cycling of proline and P5C though PRODH and the PYCR enzymes could help to provide redox balance (*33*, *34*). An increase in the level of D_6_-proline would show D_7_-proline had been oxidized by PRODH and then re-reduced by one of the PYCR enzymes (fig. S3E). However, using three different cell lines, we did not detect an increase in the level of D_6_-proline in 3D, and the ratio of D_6_-proline:D_7_-proline was the same in all samples, including the medium before it was put onto cells, indicating that no D_7_-proline had been cycled through P5C and back to proline (fig. S3, F and G). Together, these data suggest that proline accumulation in 3D is not the result of changes in proline recycling or the use of proline for ornithine or glutamate synthesis. However, it remains possible that PRODH up-regulation underpins proline accumulation in 3D cells through an unidentified mechanism.

**Fig. 4.**
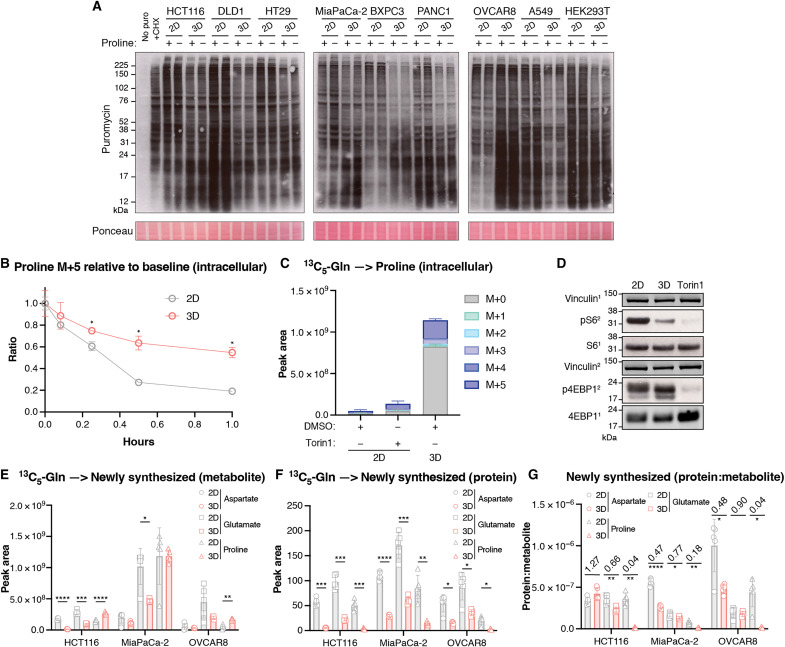
Proline incorporation into proteins is reduced in 3D culture. (**A**) Cell lines were grown in 2D or 3D in DMEM+AA+P or DMEM+AA-P. Puromycin (puro) (90 μM) was added to cell culture media 10 min before protein lysates were harvested, except where indicated. Cycloheximide (CHX), an inhibitor of translation, was added where indicated at 10 μg/ml 5 hours before harvest. Western blots show peptide puromycin incorporation. Ponceau is the loading control. (**B**) Intracellular M+5 proline in HCT116 P5CS-KO cells cultured in 2D or 3D in DMEM+AA+P with ^13^C_5_-proline at specific time points after media were changed to DMEM+AA+P with unlabeled proline, relative to the first time point. Multiple unpaired Welch *t* tests. *N* = 3. (**C**) Intracellular proline in HCT116 cells cultured in 2D with or without 250 nM Torin-1 or in 3D in DMEM+AA-P with ^13^C_5_-glutamine. *n* = 3. (**D**) Western blot showing the expression of the indicated proteins in HCT116 cells cultured in 2D with or without 250 nM Torin-1 or in 3D in DMEM+AA-P. Vinculin is the loading control. The numbers after each protein refer to the membrane the antibody was incubated with. (**E** to **G**) HCT116, MiaPaCa-2, and OVCAR8 cells were cultured in 2D or 3D in DMEM+AA-P with ^13^C_5_-glutamine before metabolites were extracted and proteins hydrolyzed. *N* = 5 technical replicates. (E) Intracellular aspartate M+4, glutamate M+5, and proline M+5 levels. (F) Protein aspartate M+4, glutamate M+5, and proline M+5 levels, extracted from protein and normalized for cell number. (G) The ratio of the level of newly synthesized amino acid in protein (protein) to the level of the same free newly synthesized amino acid (metabolite). All data are displayed as means ± SD. [(B) to (D)] Representative of at least two experiments. [(E) to (G)] Multiple unpaired Welch *t* tests corrected for multiple comparisons. (*P < 0.05, **P < 0.01, ***P < 0.001, ****P < 0.0001.)

To assess whether the reduction in cell proliferation was accompanied by changes in global protein synthesis, we examined the incorporation of puromycin into newly synthesized polypeptides. This approach showed that incorporation of puromycin into higher–molecular weight polypeptides was decreased in cells grown in 3D, with an increase in labeling of lower–molecular weight peptides in HCT116, HT29, MiaPaCa-2, BXPC3, OVCAR8, and HEK293T cells (Fig. 4A). As shown previously, levels of hyperphosphorylated 4EBP1 were also reduced in most of the 3D cultured cells (fig. S4A) (*35*). Consistently, in P5CS-KO cells, used to prevent de novo proline synthesis, we found that over time, labeled intracellular proline was depleted at a slower rate in 3D compared to 2D cells (Fig. 4B), although similar amounts of labeled proline were detected in the media of cells loaded with labeled proline, suggesting that these differences did not reflect different rates of proline loss from the cells in 2D and 3D (fig. S4B). To determine whether a global reduction in translation was causing proline accumulation, we measured proline levels in 2D cells cultured in the presence of Torin-1, a mammalian target of the rapamycin inhibitor, but this only led to a modest accumulation of proline (Fig. 4, C and D). While a general reduction in protein synthesis would not be expected to lead to a selective accumulation of proline, we considered whether there may be a differential in the use of different amino acids in 3D cells. We therefore set out to compare the levels of proline, aspartate, and glutamate incorporation into protein in 2D and 3D cells. In each of the cell lines tested, free aspartate, glutamate, and proline newly synthesized from ^13^C_5_-glutamine could be detected in both 2D and 3D cultured cells (Fig. 4E). Protein from detached cells showed a decrease in amounts of labeled proline, aspartate, and glutamate (consistent with the decreased rate of protein synthesis) (Fig. 4F). However, the fold change from 2D to 3D in the ratio of the level of newly synthesized amino acid in protein to the level of the same free newly synthesized amino acid was much smaller for proline than for glutamate or aspartate in each cell line tested, suggesting a selectively lower use of proline for general protein synthesis, potentially contributing to the accumulation of proline (Fig. 4G).

**Fig. 5.**
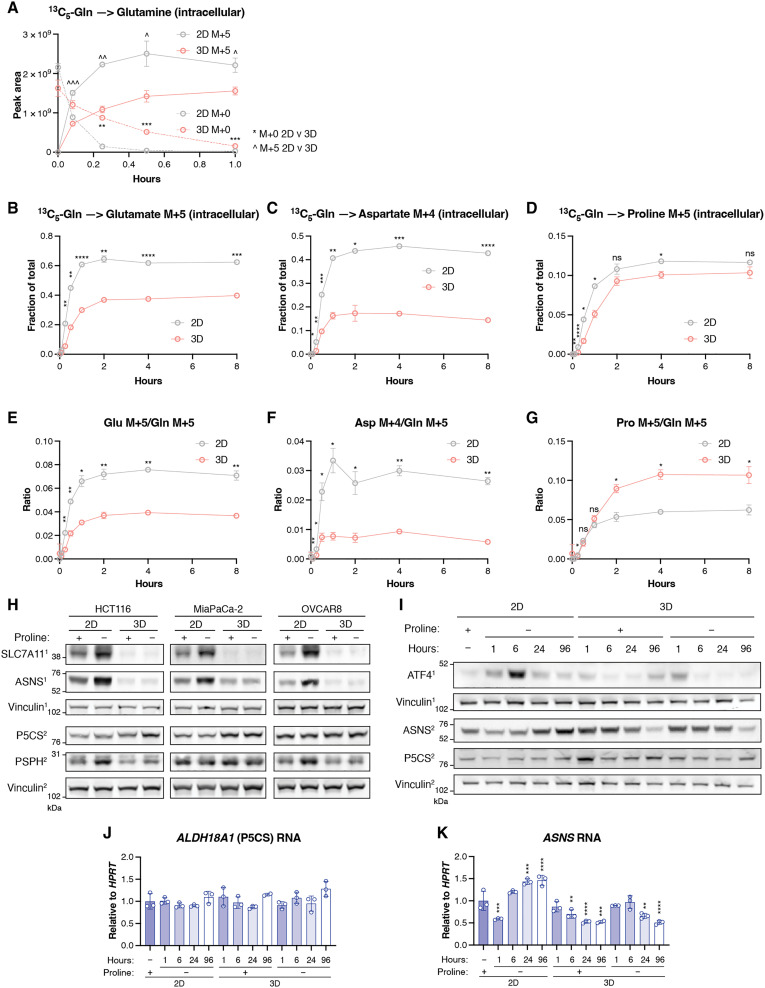
General amino acid synthesis, but not proline synthesis, drops in 3D culture. (**A** to **G**) HCT116 cells were cultured in 2D or 3D in DMEM+AA+P, and after addition of ^13^C_5_-glutamine, intracellular metabolites were analyzed at defined time points. Intracellular (A) M+0 and M+5 glutamine over 1 hour. Fraction of the total pool of (B) M+5 glutamate, (C) M+4 aspartate, and (D) M+5 proline over 8 hours. Ratio of intracellular (E) glutamate M+5, (F) aspartate M+4, and (G) proline M+5, to M+5 glutamine. *N* = 3 technical replicates at each time point. (**H**) Western blot showing the expression of the indicated proteins in HCT116, MiaPaCa-2, and OVCAR8 cells cultured in 2D and 3D in DMEM+AA+P or DMEM+AA-P. Vinculin is the loading control. (**I** to **K**) HCT116 NTC cells were cultured in 2D in DMEM+AA+P and then either moved to DMEM+AA-P or reseeded in 3D in DMEM+AA+P or DMEM+AA-P. Protein and RNA lysates were taken at the indicated time points. (I) Western blot shows the expression of the indicated proteins. Vinculin is the loading control. Relative gene expression of (J) *ALDH18A1* and (K) *ASNS* measured using quantitative polymerase chain reaction. *HPRT* was the control gene. Normalized to cells cultured in 2D with proline. *N* = 3. For Western blots, the numbers after each protein refer to the membrane the antibody was incubated with. All data representative of two experiments. Data shown as means ± SD. [(A) to (G)] Multiple unpaired Welch *t* tests, corrected for multiple comparisons. [(J) and (K)] Ordinary one-way ANOVA, comparisons to 2D + proline, corrected for multiple comparisons. [*P < 0.05, **P < 0.01, ***P < 0.001, ****P < 0.0001; in (A) * for M+0 comparison, ^ for M+5 comparison.]

Having established a disproportionate reduction in proline utilization in 3D cells, we examined whether there was a change in the rate of newly synthesized proline accumulation in response to detachment. Consistent with slower growth and rates of protein synthesis, detached cells showed a decreased uptake of labeled glutamine, slower consumption of unlabeled glutamine following glutamine labeling (Fig. 5A and fig. S5, A and B), and decreased net synthesis of glutamate and aspartate that was evident over the time taken to reach steady state (Fig. 5, B and C). However, we noted that net synthesis of proline was not decreased in detached cells (Fig. 5D). Furthermore, when compared to glutamine uptake, the net rate of proline synthesis was maintained in detached cells, while net rate of aspartate and glutamate synthesis showed a relative decrease (Fig. 5, E to G). These results suggest a retention of proline production in 3D cells compared to other amino acids and a selective channeling of available glutamine into proline. The expression of enzymes required for the de novo synthesis of many amino acids is controlled by activating transcription factor 4 (ATF4), a transcription factor that responds to nutrient starvation. We found that detachment resulted in a decreased production of asparagine synthetase (ASNS) (required for asparagine synthesis) and phosphoserine phosphatase (PSPH) (required for serine synthesis), along with a clear down-regulation of the transporter SLC7A11, another well-established ATF4 target (*36*, *37*) (Fig. 5H). However, as also shown earlier (Fig. 1H), there was no decrease in expression of the proline synthesis pathway enzymes, although both *PYCR1* and *P5CS* are regulated by ATF4 in mammalian cells (*36*). After the removal of proline from the tissue culture media, the level of ATF4 protein in HCT116 cells was elevated in 2D cells after 6 hours, while cells cultured without proline in 3D for the same duration failed to show this increase and displayed a slight drop in ATF4 levels over 24 hours (Fig. 5I). Proline does not begin to accumulate in cells cultured in 3D for at least after 24 hours after detachment, indicating that the lack of ATF4 response in 3D cultured cells is not due to the presence of proline (fig. S5C). Reverse transcription polymerase chain reaction analysis showed that while the level of *ALDH18A1* mRNA (encoding P5CS) did not change over 24 hours of proline starvation in 2D or 3D culture, *ASNS* mRNA levels increased in response to proline withdrawal in 2D but dropped over time in 3D culture, regardless of proline availability (Fig. 5, J and K). These results suggest that while there is a general dampening in ATF4 signaling in 3D, proline synthesis enzymes are maintained through ATF4-independent mechanisms relative to enzymes that produce other amino acids in detached cells, making proline exempt from the general down-regulation of amino acid synthesis in slower-growing cells.

**Fig. 6.**
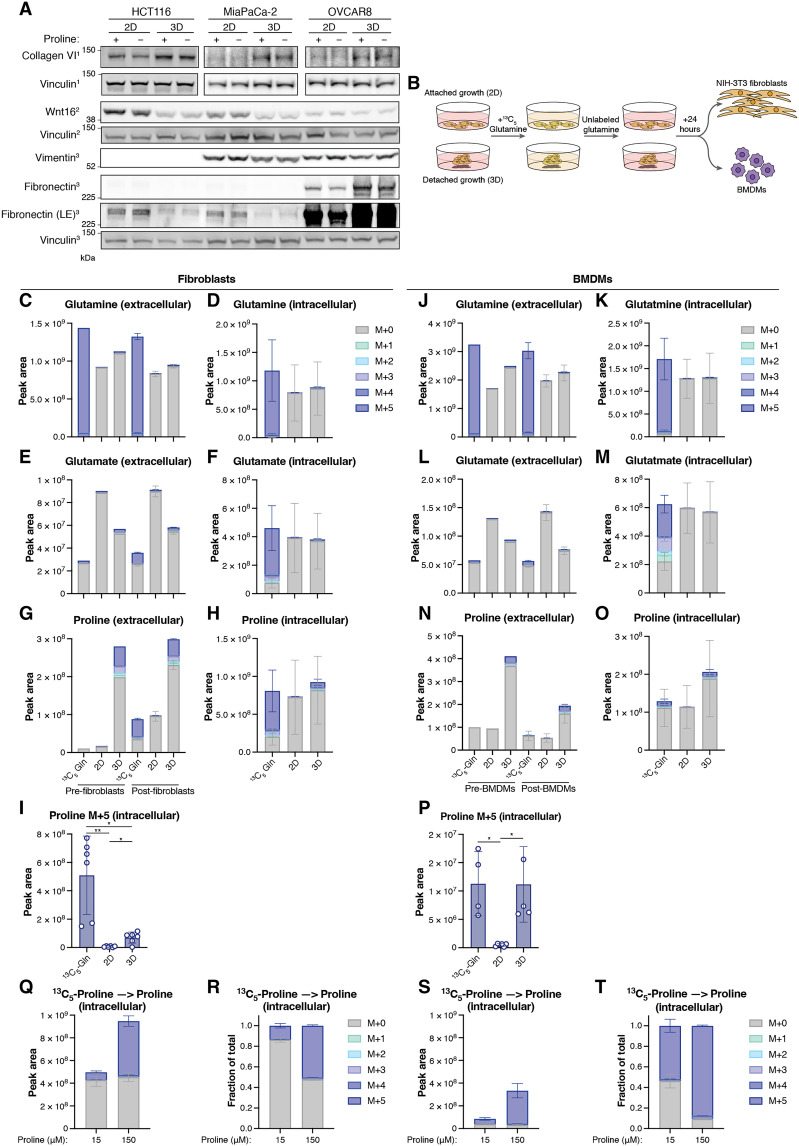
Macrophages take up proline from 3D cultured cells. (**A**) Western blot showing levels of the indicated proteins in HCT116, MiaPaCa-2, and OVCAR8 cells cultured in 2D and 3D in DMEM+AA+P or DMEM+AA-P. Vinculin is the loading control. LE, long exposure. (**B**) Experiment schematic. HCT116 cells growing in 2D or 3D culture were cultured in media containing ^13^C_5_-glutamine. Media were replaced with media containing unlabeled glutamine for 24 hours and then put onto NIH-3 T3 immortalized fibroblasts or bone marrow–derived macrophages (BMDMs) for 7 hours. Alternatively, BMDMs and fibroblasts were given fresh DMEM+AA-P with ^13^C_5_-glutamine. (**C**) Levels of glutamine in the different media before (Pre, *n* = 1) and after (Post, *n* = 6) they were given to fibroblasts. (**D**) Fibroblast intracellular glutamine. (**E**) As in (C) for glutamate. (**F**) Fibroblast intracellular glutamate. (**G**) As in (C) for proline. (**H**) Fibroblast intracellular proline. (**I**) Fibroblast intracellular proline M+5. [(C) to (I)] *n* = 6 technical replicates. (**J**) Levels of glutamine in the different media before (Pre, *n* = 1) and after (Post, *n* = 6) they were given to BMDMs. (**K**) BMDM intracellular glutamine. (**L**) As in (J) for glutamate. (**M**) BMDM intracellular glutamate. (**N**) As in (J) for proline. (**O**) BMDM intracellular proline. (**P**) BMDM intracellular proline M+5. [(J) to (P)] *n* ≥ 4 technical replicates. (**Q** to **T**) Fibroblasts and macrophages were cultured in DMEM+AA+P with either 15 or 150 μM proline. Six hours after the media were changed to media containing ^13^C_5_-proline at the same concentration, metabolites were extracted. (Q) Intracellular levels of proline and (R) distribution of proline isotopologs as a fraction of total intracellular proline in fibroblasts. (S) Intracellular levels of proline and (T) distribution of proline isotopologs as a fraction of total intracellular proline in macrophages. All metabolites measured using LC-MS. [(C) to (P)] Data are shown as means ± SD. [(I) and (P)] Brown-Forsythe and Welch ANOVA test (*P < 0.05, **P < 0.01; unpaired *t* tests). Three outliers removed.

A major use of proline in the cell is to synthesize collagen, matrix proteins that provide support for detached cells. Despite the general reduction in protein synthesis and the selectively lower use of proline in protein synthesis, cells grown in 3D showed increased expression of collagen VI, suggesting that collagen production may be protected in these cells, potentially to allow adhesion and survival (Fig. 6A). This was apparently not part of a general increase in the expression of secreted or structural proteins as Wnt16 and fibronectin levels did not consistently increase in cells cultured in 3D compared to cells cultured in 2D across cell lines (Fig. 6A).

Our tissue culture experiments also showed that many cancer cells excrete proline, and we considered whether proline accumulation and excretion by detached cancer cells could be used by other cells in the TME. Fibroblasts in the TME are the principal producers of collagen and have a high demand for proline (*38*). We therefore examined whether fibroblasts use cancer cell–produced proline. Either media containing labeled glutamine (^13^C_5_-Gln) or media conditioned by attached or detached HCT116 cells that had been preincubated with labeled glutamine (to allow for the synthesis of labeled proline) was provided to immortalized fibroblasts (NIH-3 T3) (Fig. 6B). We confirmed that almost no labeled glutamine or glutamate remained in the medium conditioned by HCT116 cells (Fig. 6, C and E), leading to very low levels of labeled intracellular glutamine and glutamate in fibroblasts grown in this conditioned medium (Fig. 6, D and F). As previously seen, labeled proline synthesized by HCT116 cells was more abundant in media conditioned by 3D cells than 2D cells (Fig. 6G). As expected, cells grown in labeled glutamine showed substantial labeling of intracellular glutamine and glutamate (Fig. 6, D and F), which was used to synthesize proline—more than 50% of the intracellular proline was derived from labeled glutamine (Fig. 6H). However, these cells showed only minimal uptake of exogenous labeled proline from media conditioned by HCT116 2D or 3D cells (Fig. 6H) and in fact total extracellular proline in the ^13^C_5_-Gln media, and the 2D and 3D conditioned media increased following incubation with the fibroblasts (Fig. 6G). These observations suggest that fibroblasts can efficiently provide their own proline through de novo synthesis, rather than taking up exogenous proline.

Another key component of the TME are macrophages, and polarization to an inflammatory M2-like state is associated with enhanced tumor-promoting activity of these cells. M2-polarized macrophages efficiently degrade and consume collagen (*39*) and proline metabolism has been implicated in M2 macrophage polarization (*40*). Repeating the fibroblast studies using bone marrow–derived macrophages (BMDMs), we reproduced the lack of extracellular or intracellular label in glutamine or glutamate in cells exposed to HCT116-conditioned medium (Fig. 6, J to M). However, these cells depleted extracellular proline from the medium conditioned by 3D cancer cells (Fig. 6N), and showed an increase in intracellular proline (Fig. 6O). These cells also showed evidence of proline synthesis from labeled glutamine, although this represented only a small fraction of all proline (~10%) (Fig. 6O). BMDMs therefore use both de novo synthesized and imported proline and can access proline produced by cancer cells.

Using these data, we assessed the levels of M+5 labeled intracellular proline derived either from glutamine (through de novo synthesis) or from 3D HCT116-conditioned medium (through uptake of extracellular proline produced by the cancer cells) (Fig. 6, I and P). These data show that the proportion of proline synthesized by the cell from glutamine is much higher in fibroblasts than in macrophages, where labeled proline derived from 3D cell–conditioned medium was similar to that synthesized de novo from glutamine.

To more clearly determine the differences in proline uptake and metabolism between fibroblasts and macrophages, we fed both cell types two different concentrations of labeled proline (15 and 150 μM). Both fibroblasts and macrophages take up proline from the media, but the fraction of unlabeled proline was much greater at each concentration in fibroblasts, suggesting that they synthesize a greater proportion of their proline than macrophages, which contain a greater proportion of externally derived (labeled) proline (Fig. 6, Q to T). Together, these results suggest that the dependence of different cells in the TME on exogenous proline may differ, with macrophages showing a higher requirement than fibroblasts. Proline secretion by cancer cells may therefore have variable effects on the composition of the TME.

## DISCUSSION

We show here that many cancer cells respond to detachment by selectively accumulating and secreting proline. The accumulation of metabolites can be the result of two mutually compatible responses—increased synthesis and/or decreased utilization. Our data show that both effects play a role in the accumulation of proline in cells following detachment. Loss of attachment leads to a decrease in the proliferation rate of the cells, with detached cells showing slower G_1_ progression than attached cells. Accompanying this reduction in growth rate is a relative reduction in overall protein synthesis in detached cells. Our data indicate that proline levels may be maintained during detachment in part by being selectively less incorporated into all proteins compared to glutamine (although we noted a selective retention of proline-rich collagen production in 3D; see below), allowing for a relative accumulation of proline. Another response to slower growth is a decrease in expression of the enzymes involved in the de novo synthesis of amino acids such as ASNS and PSPH, with evidence for a general down-regulation of ATF4 target genes. This response would coordinate with the lower demand for amino acids due to a slowing in protein synthesis. Exceptionally, expression of the enzymes of the proline synthesis pathway are protected from this down-regulation of expression, despite also being transcriptionally regulated by ATF4. Maintenance of proline synthesis under conditions of reduced growth rate and protein synthesis would also result in proline accumulation. Further studies are required to understand the mechanisms underlying this selective retention of proline synthesis enzyme expression.

Proline metabolism has been shown to promote cancer development and progression by different mechanisms. Redox imbalance resulting from cancer-associated stress such as hypoxia and mitochondrial disfunction is ameliorated in tumor cells by the up-regulation of oxygen independent pathways of NAD^+^ recycling such as lactate dehydrogenase–mediated lactate production (*41*, *42*). Mitochondrial proline synthesis achieves a similar goal and has been shown to be important under hypoxia (*18*). Previous work has shown that detached cells also induce redox balancing pathways, raising the possibility that the retention of proline synthesis in these cells contributes to a balanced NAD^+^/NADH ratio (*1*). However, we were unable to detect a defect in the growth of detached cells depleted of P5CS, which is essential for the first steps of proline synthesis under these growth conditions. These results indicate that other pathways for NAD^+^ regeneration are sufficient to allow optimal cell growth and survival in tissue culture in the absence of proline synthesis, although different demands on cancer cells in vivo may increase the dependence on proline synthesis for redox balance. Proline synthesis is dependent on NADPH (*28*, *43*) and increased activity of this pathway could deplete antioxidant defense capacity and increase the vulnerability of cells to forms of cell death such as ferroptosis.

Production of the proline-rich extracellular matrix protein collagen by cancer cells plays an important role in supporting cancer cell metastasis (*44*, *45*). Previous studies have shown that increased proline synthesis by cancer cells can promote tumorigenesis (*17*, *46*), and we note that despite an overall reduction in proline accumulation into proteins, collagen VI expression is increased in detached cells. It is possible that the maintenance of proline synthesis and collagen production under detached conditions is contributing to the survival of these cells, potentially by allowing the retention of some degree of matrix support. However, the increased proline accumulation in detached cells is also accompanied by increased excretion of proline, leading to the accumulation of proline in the medium. Turning to components of the TME, we showed that macrophages, but not fibroblasts, take up tumor cell synthesized proline despite being competent to produce proline through the de novo synthesis pathway. Cancer-associated fibroblasts increase in PYCR1 expression and proline synthesis for collagen production, highlighting the importance of proline in the establishment of a tumor-supportive environment (*38*).

The role of proline production and catabolism in cancer is complex and appears to be tissue dependent, with studies showing both tumor-supportive and tumor-suppressive roles for enzymes such as PRODH (*32*, *46*, *47*). While we have confirmed previous studies showing that transformed breast epithelial cells grown as spheroids in soft agar accumulate less endogenous proline (fig. S1C) (*23*), we find here that the same cells grown under detachment (in the absence of exogenous proline) contain higher levels of proline. This therefore suggests that culture conditions can affect proline metabolism. However, while many of the cell lines we tested accumulated and excreted proline under detached conditions, three of them (PANC1, BXPC3, and ASPC1) show reduced extracellular proline accumulation in detached compared to attached conditions (Fig. 1), suggesting that the response also shows some cell-type dependence. While the previous study also illuminated the possible role of proline recycling in maintaining cell viability, we were unable to identify proline recycling in the model of detachment described here. Further studies will be necessary to determine why these different models produce opposing effects on proline accumulation and how this affects tumor progression in vivo.

Last, as seen in the previous publication, we detected a strong up-regulation of PRODH expression in almost all the detached cells. Although we were unable to ascribe a selective role for this reaction during detachment in our model system, we note that these data are consistent with a role for proline catabolism by PRODH under detached conditions.

## MATERIALS AND METHODS

### Cell culture

All cell lines used in this study were obtained from the Crick Cell Services except MCF10A cells, which were obtained from American Type Culture Collection. Mycoplasma screening and validation using short tandem repeat profiling were also performed by Crick Cell Services, and MCF10A cells were mycoplasma tested in the Fendt laboratory. Cells were cultured in DMEM (Gibco, 41966) with 10% fetal bovine serum and 1% penicillin/streptomycin and supplemented with the following nonessential amino acids (DMEM+AA+P): l-alanine (150 μM), l-aspartic acid (150 μM), l-asparagine (340 μM) l-glutamic acid (150 μM), and l-proline (150 μM). Where indicated, proline was removed (DMEM+AA-P). For labeling experiments or experiments requiring proline starvation, DMEM+AA-P with dialyzed fetal bovine serum (FBS) (Gibco) was used.

Detached cells were grown in six-well ultralow attachment plates (Corning). Unless otherwise stated, cells were seeded at 2 × 10^5^ cells per well in 2 ml of media. To change the media, plates were tilted and left until clusters settled at the bottom of the well before media was removed using an aspirator or pipette and then refilled. Cells were grown in 3D conditions for at least 4 days before analysis.

MCF10A-HRAS^V12^ cells were generated and cultured as previously described (*23*). Briefly, these cells were maintained in DMEM-F12 supplemented with 5% horse serum, 1% penicillin/streptomycin, hydrocortisone (0.5 mg/ml), cholera toxin (100 ng/ml), insulin (10 mg/ml), and recombinant human epidermal growth factor (20 ng/ml). In soft agar culture, 1% soft agar in water was mixed 1:1 with prewarmed media and 3 ml added into wells of six-well plates. After plates solidified, 1.2 × 10^4^ cells were plated on top and incubated for 5 days. For experiments, the cells were transferred to DMEM+AA-P or DMEM+AA+P, as required.

### P5CS deletion

Plasmids containing the Cas9 gene and either nontargeting control (NTC) or *ALDH18A1* (encoding P5CS) targeting single-guide RNAs were purchased from Santa Cruz Biotechnology, along with the corresponding homology-directed repair (HDR) plasmids. A total of 1.5 × 10^5^ HCT116 cells were transfected in DMEM+AA+P with 1 μg each of *ALDH18A1* CRISPR and HDR plasmids, or 1 μg of the NTC plasmid using JetPrime (PolyPlus). Successful expression of the Cas9 plasmid was confirmed by the observation of green fluorescent protein using a fluorescence microscope. After expansion of the cultures, puromycin (1 μg/ml, Sigma-Aldrich) was added to select for cells with successful HDR. To obtain single cell clones, wells of a 96-well plate were filled with 200 μl of a suspension containing 2.5 cells/ml. After clones had expanded, protein extracts were taken in radioimmunoprecipitation assay (RIPA) lysis buffer and Western blotting used to detect the presence of the P5CS enzyme. Twenty-three clones in which P5CS had successfully been deleted were pooled.

### siRNA knockdown

siRNAs were diluted in OptiMEM (Gibco) and Lipofectamine RNAiMax before the mixture was added to the cell media at a final concentration of 20 nM. Cells were incubated in the transfection mixture for 24 hours before media was replaced. All siRNAs used in this study were “ON-TARGET plus” from Horizon: NTC (001810), *PYCR1* (012349), *PYCR2* (016646), *PYCRL* (014246), *ALDH18A1* (006785), and *PRODH* (009543).

### Proliferation assays

Cells were seeded at 1 × 10^4^ cells per well in 24-well plates. The day after plating, the media was replaced with the test media. For counting, cells were washed and harvested with trypsin. After quenching with media containing FBS, 400 μl of the cell solution was added to 19.6 ml of CASYton solution and analyzed with a CASY cell counted to determine cells per milliliter. Alternatively, CellTiter-Glo (Promega) assay solution was used according to the manufacturer’s instructions. Cells were seeded at 2 × 10^3^ cells per well in 96-well plates. Briefly, half the cell media was removed, and the CellTiter-Glo assay solution was added to the well to a 1:1 ratio with the remaining cell media. The solution was pipetted up and down until cell lysis was observed. After 20-min equilibration, luminescence was measured using a PHERAstar plate reader (BMG Labtech).

### si*PRODH* proliferation assay

HCT116 cells expressing near-infrared expressing protein (HCT116-iRFP) as previously described in (*48*) were seeded in 96-well black 2D plates (Corning) or 96-well black ultralow attachment round-bottom plates (Corning) and transfected with NTC siRNA or an siRNA targeting *PRODH*. Each subsequent day, plates were scanned using an Odyssey scanner (Li-Cor) to measure iRFP fluorescence, used as described previously (*49*) as a proxy for cell number. Plates were scanned at 169-μm resolution with a 3.5-mm offset at a low-intensity setting. The fluorescence in each well was quantified using Image Studio software (Li-Cor, V5.2).

### Cell cycle analysis

Cells were trypsinized and harvested in media containing FBS, spun down, and washed in phosphate-buffered saline (PBS). Cold 70% ethanol was then added dropwise while vortexing before incubating on ice for at least 30 min. Cells were washed three times in cold PBS before staining in 1:10,000 propidium iodide plus RNase A (10 μg/ml) at 37°C for at least 30 min. Data were collected by flow cytometry, ensuring the event rate did not exceed 400 events/s. Cell cycle profiles were analyzed in FlowJo using the Watson model or by manually setting the gating.

### Double thymidine block

To synchronize cells at the G_1_-S checkpoint, 2 mM thymidine (Sigma-Aldrich) was added to exponentially growing cells. After 16 hours, cells were washed three times in PBS and fresh media added. After 6 hours, 2 mM thymidine was added to the cells for another 16 hours before harvesting for cell cycle analysis. For some samples, after the second incubation in thymidine, cells were washed three times in PBS and fresh media added before harvesting a few hours later.

### Western blotting

After washing with PBS, cell proteins were extracted using 10% RIPA lysis buffer (Millipore) with 0.1% SDS, protease inhibitors (Roche), and phosphatase inhibitors (Thermo Fisher Scientific). Extracts were centrifuged at 21,100*g* for 10 min at 4°C and the supernatant retained. Protein concentration was determined using a bicinchoninic acid (BCA) protein assay (Thermo Fisher Scientific) and then normalized in RIPA buffer and NuPAGE LDS sample buffer (Life Technologies) with 5% 2-mercaptoethanol, before samples were heated to 75°C in for 10 min. After electrophoresis at 160 V (or lower) in MES or Mops buffer, proteins were transferred to nitrocellulose membranes (Amersham) in tris-glycine-methanol buffer at 300 mA for 2 hours at 4°C. Membranes were blocked with 5% bovine serum albumin (BSA) (Sigma-Aldrich) in tris-buffered saline with 0.5% Tween 20 (TBST) buffer for at least 1 hour before overnight incubation with primary antibodies at 4°C. A list of antibodies and the concentrations used in this study are as follows: vinculin (sc-73614, 1:1000, Santa Cruz Biotechnology), P5CS (17719-1-AP, 1:1000, Proteintech), PYCR1 (13108-1-AP, 1:1000, Proteintech), PYCR2 (17146-1-AP, 1:2000, Proteintech), PYCRL (MA5-25320, 1:2000, Thermo Fisher Scientific), PRODH (sc-376401, 1:500, Santa Cruz Biotechnology), p53 [DO-1, 1:2000, (*50*)], actin (13E5, 1:2000, Cell Signaling Technology), puromycin (MABE343 12D10, 1:20,000, Millipore), p21 (sc-397, 1:200, Santa Cruz Biotechnology), SLC7A11 (D2M7A, 1:1000, Cell Signaling Technology), ASNS (HPA029318, 1:1000, Atlas Antibodies), PSPH (ab211418, 1:1000, Abcam), S6 (54D2, 1:1000, Cell Signaling Technology), p-S6 S235/236 (2211, 1:1000, Cell Signaling Technology), 4EBP1 (53H11, 1:1000, Cell Signaling Technology), p-4EBP1 T37/46 (236B4, 1:1000, Cell Signaling Technology), collagen VI (ab182744, 1:1000, Abcam), Wnt16 (sc-271897, 1:200, Santa Cruz Biotechnology), fibronectin (sc-18825, 1:1000, Santa Cruz Biotechnology), vimentin (ab92547, 1:10,000, Abcam), and glyceraldehyde-3-phosphate dehydrogenase (sc-32233, 1:1000, Santa Cruz Biotechnology). Blots were washed three times in TBST before appropriate secondary antibodies were added in 5% BSA-TBST for 1 hour. After three more washes with TBST, membrane-bound antibodies were detected using either an Odyssey scanner (Li-Cor) and analyzed with Image Studio software (Li-Cor) or chemiluminescence (Pierce).

### Puromycin incorporation assay

Except for the negative control sample, 90 μM puromycin (Sigma-Aldrich) was added into the media of cells that were approximately 50% confluent. After 10-min incubation, cells were washed with cold PBS and then lysed in RIPA buffer. Five hours before adding puromycin, cycloheximide (10 μg/ml; Sigma-Aldrich) was added to one well to inhibit translation. Western blots were probed with an anti-puromycin antibody to analyze puromycin incorporation.

### Metabolite extraction

On the day of extraction, media were replaced with media containing a labeled compound (^13^C_5_-glutamine 2 mM or ^13^C_5_-proline 150 μM, both Sigma-Aldrich). After steady state was reached (unless stated otherwise) 10 μl of the cell supernatant was diluted in 490 μl of metabolite extraction buffer (MEB; 50% methanol, 30% acetonitrile, 20% water). Samples from 2D cultures were extracted from cells at <80% confluence. Cells growing in 2D were washed in cold PBS and lysed in cold MEB on ice. Cells growing in 3D were allowed to settle at the bottom of cultures wells before they were washed in cold PBS and lysed in cold MEB on ice. Extracts were spun at 21,100*g* for 10 min at 4°C, and the supernatants were retained for liquid chromatography–mass spectrometry (LC-MS). Unless otherwise stated, metabolite levels were normalized by cell number at the point of extraction. Before each metabolite extraction, cells in a parallel well were counted so that the amount of MEB used could be calculated to lyse cells at 2 × 10^6^ cells/ml. Where metabolite levels were normalized by protein content, cells were extracted as previously, in 1 ml of cold MEB. Following centrifugation, the supernatants were dried under a vacuum and resuspended in MEB at 250 μg/ml. The protein content of the cells was calculated by determining the protein content of cells in a parallel well using a BCA assay as previously described. Samples were passed to the Crick Metabolomics Facility for LC-MS analysis.

### Protein hydrolysis

The media of cells growing in 2D or 3D was changed to DMEM+AA-P with ^13^C_5_-glutamine for 6 hours before the extracellular and intracellular metabolites were extracted as previously described (*51*). The nonaqueous pellet was washed in cold water and hydrolyzed in 6 M HCl for 24 hours at 95°C. The hydrolyte was neutralized with NaOH and spun down at 21,100*g* for 10 min at 4°C. The supernatant was diluted 1 in 8 in MEB for LC-MS analysis. Data were normalized to cell number.

### Liquid chromatography–mass spectrometry

Metabolite analysis was performed by LC-MS as described previously (*52*). Briefly, chromatographic separation was performed using a SeQuant Zic-pHILIC (Merck Millipore) column (5-μm particle size, polymeric, 150 mm by 4.6 mm), and metabolites were detected with an Q-EXACTIVE Plus (Orbitrap) mass spectrometer (Thermo Fisher Scientific) coupled to a Vanquish UHPLC system (Thermo Fisher Scientific). The injection volume was 5 μl, the oven temperature was maintained at 25°C, and the autosampler tray temperature was maintained at 4°C. Elution buffer A was acetonitrile and buffer B was 20 mM ammonium carbonate with 0.1% ammonium hydroxide in water. For chromatographic separation, a linear gradient was set up at a constant flow rate of 300 μl/min starting with 80% buffer A dropping to 5% over 17 min, holding at 5% of A for 3 min and lastly re-equilibrating the column at 80% of A over 4 min. MS was performed with a heated electrospray ionization (ESI) II probe and operated in full-scan mode with positive/negative polarity switching. MS parameters were as follows: spray voltage, 3.5 and 3.2 kV for positive and negative modes, respectively; probe temperature, 320°C; sheath and auxiliary gases were 30 and 5 arbitrary units, respectively; and full-scan range: 70 to 1050 mass/charge ratio (*m/z*) with settings of automated gain control target and resolution as balanced and high (3 × 10^6^ and 70,000) respectively. Data were recorded using Xcalibur 4.2.47 software (Thermo Fisher Scientific). Calmix solution (Thermo Fisher Scientific) was used for mass calibration for both polarities. Lock-mass correction was used to enhance calibration stability and applied to each analytical run using ubiquitous low-mass contaminants. Parallel reaction monitoring acquisition parameters were the following: resolution, 17,500; collision energies were set individually in high-energy collisional dissociation mode. Metabolites were identified and quantified by accurate mass and retention time and by comparison to the retention times, mass spectra, and responses of known amounts of authentic standards using TraceFinder 4.1 EFS software (Thermo Fisher Scientific). Label incorporation and abundance was estimated using TraceFinder 4.1 EFS software. The level of labeling of individual metabolites was estimated as the percentage of the metabolite pool containing one or more ^13^C atoms after correction for natural abundance isotopes (*53*).

### Gas chromatography–mass spectrometry

Proline was measured with gas chromatography–mass spectrometry. For this, the samples were extracted and derivatized as previously described (*23*, *54*). In brief, polar metabolites were derivatized at 37°C with 20 μl of methoxyamine (20 mg/ml) in pyridine for an hour and half. Next, 15 μl of *N*-(tert-butyldimethylsilyl)-*N*-methyl-trifluoroacetamide, with 1% tert-butyldimethylchlorosilane was added to 7.5 μl of each sample and incubated for 1 hour at 60°C. Isotopolog distributions and metabolite concentrations were measured with a 7890 A gas chromatography (GC) system combined with a 5975C Inert MS system (Agilent Technologies). One microliter of sample was injected into a DB35MS column in splitless mode using an inlet temperature of 270°C. The carrier gas was helium with a flow rate of 1 ml/min. Upon injection, the GC oven was set at 100°C for 1 min and then increased to 105° at 2.5°C/min and with a gradient of 2.5°C/min lastly to 320° at 22°C/min. Isotopolog distributions and peak areas were extracted from the raw ion chromatograms using an in-house Matlab script, which applies consistent integration bounds and baseline correction to each ion. In addition, we corrected for naturally occurring isotopes and normalized peak areas to the protein content of the sample and internal standard glutaric acid.

### Liquid chromatography–tandem MS

For detection of NAD^+^ and NADH, liquid chromatography–tandem MS was used. Data acquisition was performed using an adaptation of a method previously described. Samples were injected into using an Dionex UltiMate 3000 LC system (Thermo Fisher Scientific) with a ZIC-pHILIC (150 mm by 4.6 mm, 5-μm particle) column (Merck Sequant). A 15-min elution gradient of 80% solvent A to 20% solvent B was used, followed by a 5-min wash of 95:5 solvent A to solvent B and 5-min re-equilibration, where sSolvent B was acetonitrile (Optima HPLC grade, Sigma-Aldrich) and solvent A was 20 mM ammonium carbonate in water (Optima HPLC grade, Sigma-Aldrich). Other parameters were as follows: flow rate, 300 μl/min; column temperature, 25°C; injection volume, 10 μl; autosampler temperature, 4°C. MS was performed in positive polarity using a TQS Quantiva Triple Quadrupole Mass Spectrometer (Thermo Fisher Scientific) with an ESI source. Qualitative and quantitative analysis was performed using Xcalibur Qual Browser and TraceFinder 4.1 software (Thermo Fisher Scientific) according to the manufacturer’s workflows. Analyses were performed in selected reaction monitoring. Precursor to product ions transitions and collision energies are listed in Table 1.

**Table 1.** Precursor to product ion transitions and collision energies.

Compound	Precursor (*m/z*)	Product (*m/z*)	Collision energy (V)
NADH	666.132	514.051	22
		649	20
Proline	116.071	70.058	16.17
		70.065	17
D_7_-proline	123.071	77.058	16.17
		77.065	17
NAD	664.116	136.062	10
		428.036	25

### Quantitative polymerase chain reaction

Total RNA from HCT116 NTC cells was extracted using RNeasy Mini kit (Qiagen) with on-column DNA digestion (Qiagen, RNase-Free DNase Set). The High-Capacity cDNA Reverse Transcription kit (Thermo Fisher Scientific, catalog no. 4368814) was used to synthesise cDNA according to the manufacturer’s instructions. Quantitative polymerase chain reaction (qPCR) was performed using SYBR Green qPCR Master Mix (Sigma-Aldrich) with the PrimeTime qPCR primers (IDT; *ASNS* Hs.PT.56a.28032225, *ALDH18A1* Hs.PT.58.15308001, *HPRT* Hs.PT.58v.45621572). The QuantStudio 7 Flex Real-Time PCR System (software v1.3) was used for all reactions. Gene expression was normalized to HPRT housekeeper gene, analyzed according to Pfaffl method (*55*) and expressed as relative units.

### Macrophage extraction and differentiation

A syringe filled with PBS and 25G needle was used to flush the bone marrow out of a femur from a C57Bl/6 mouse. After red blood cells lysis, the remaining cells were plated in nontissue culture treated 10-cm dishes at approximately 5 × 10^6^ cells per plate with mouse recombinant macrophage colony-stimulating factor (20 ng/ml; PeproTech).

### Statistical analysis

All data are expressed as the means ± SD. Data collection was performed in Excel (version 16.64), and statistical analyses were performed in GraphPad Prism (version 9.4.1) and are described in the figure legends.
