# Supplementary material for: Loss of attachment promotes proline accumulation and excretion in cancer cells
Source: Sci Adv. 2023 Sep 6;9(36):eadh2023. doi: 10.1126/sciadv.adh2023 (PMC10482343; doi:10.1126/sciadv.adh2023)
Supplement: Supplementary file 1 — Figs. S1 to S5 [file sciadv.adh2023_sm.pdf]

Science Advances
AAAS

Supplementary Materials for

Loss of attachment promotes proline accumulation and excretion in cancer cells

Steven E. Pilley *et al.*

Corresponding author: Karen H. Vousden, karen.vousden@crick.ac.uk

Sci. Adv. **9**, eadh2023 (2023)
DOI: 10.1126/sciadv.adh2023

This PDF file includes:

Figs. S1 to S5

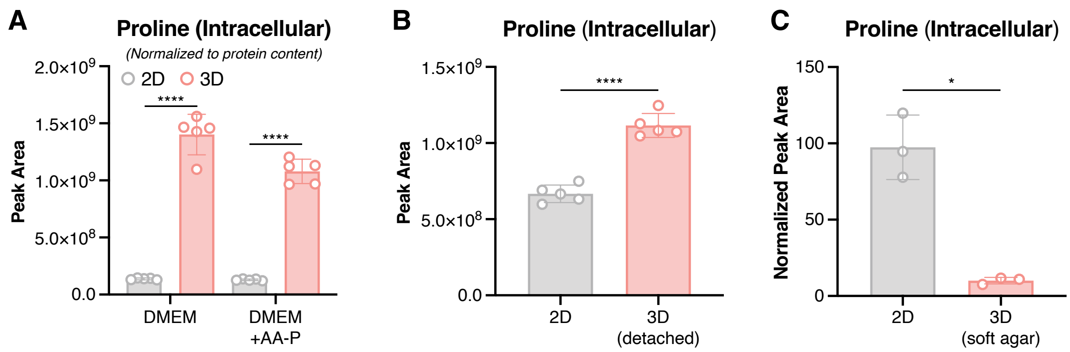

Figure S1: **Proline accumulates in cells grown in detached conditions but decreases in cells grown in soft agar.**

A) Intracellular proline levels in 2D and 3D cells incubated in the indicated media normalized to protein content. Measured using LC-MS. N = 5. B) Intracellular proline levels in MCF10A H-Ras^{V12} cells cultured in 2D and detached conditions in DMEM+AA-P. Measured using LC-MS. N = 5. C) Intracellular proline levels in MCF10A H-Ras^{V12} cells cultured as previously described in 2D and soft agar in DMEM-F12 (containing proline)*(23)*. Measured using GC-MS, normalized to internal standards and protein content. N = 3. Experiments in panels B and C were carried out independently. All data presented as mean ± SD. A) Multiple unpaired Welch t-tests corrected for multiple comparisons, B), C) Welch's t-test: * $p < 0.05$, **** $p < 0.0001$.

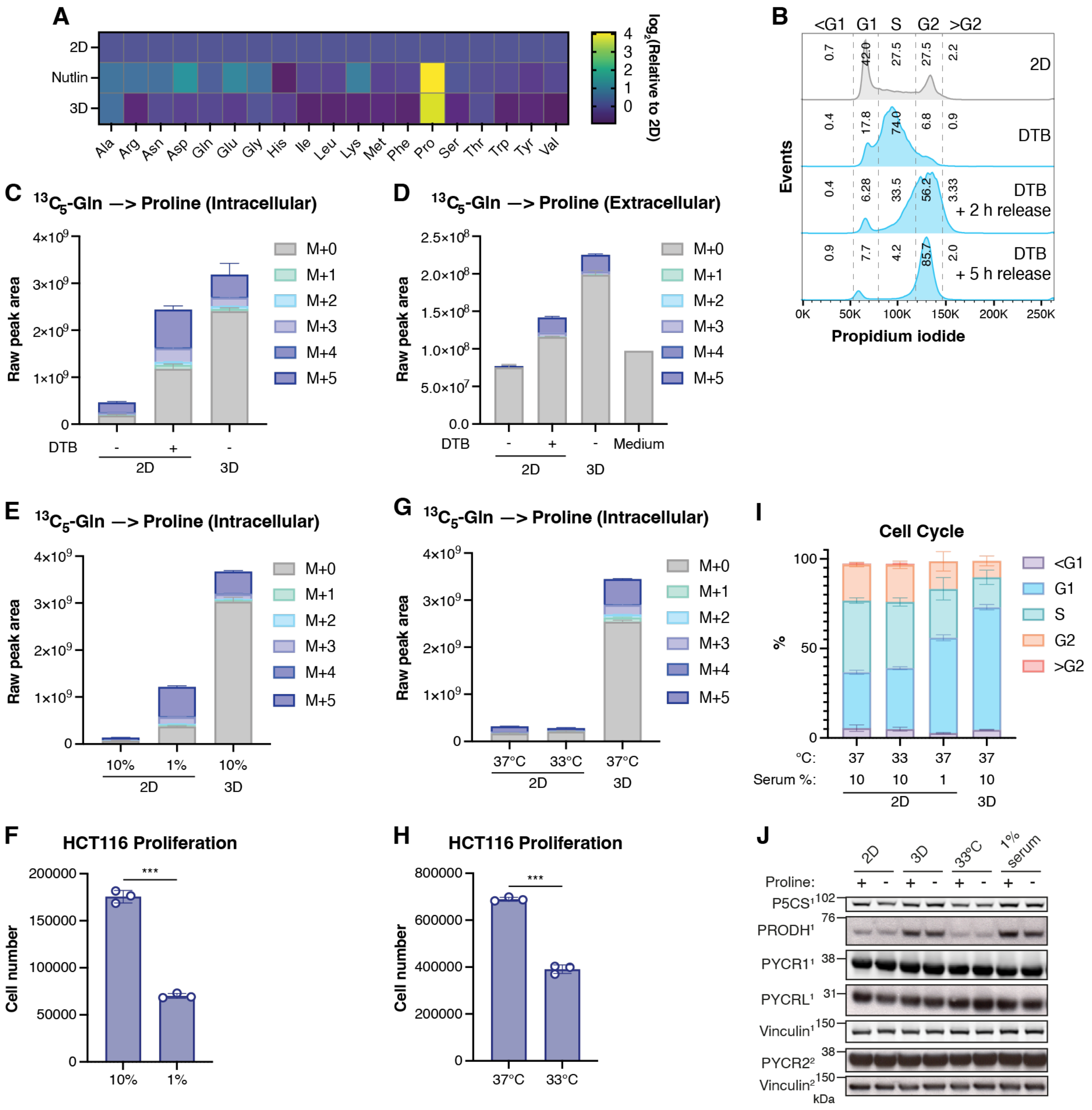

Figure S2: Cell cycle delay and not slower proliferation affects proline accumulation in 2D.

A) Heatmap of the log 2-fold change of the level of each measured intracellular amino acid in 3D and 2D-Nutlin-treated cells compared to 2D. Performed in DMEM+AA-P. B) Representative histograms of cells treated with a double thymidine block (DTB) in DMEM+AA-P stained with propidium iodide to show cell cycle distribution. Release indicates that thymidine was removed from the media allowing the cell cycle to restart. Numbers indicate the percentage of cells in each cell cycle stage. C) Intracellular and D) extracellular proline of HCT116 cells cultured in 2D treated with or without a DTB or in 3D in DMEM+AA-

P with $^{13}C_5$-glutamine. ‘Medium’ shows proline level in the medium before it was put onto cells. E) Intracellular proline in HCT116 cells cultured in 2D in DMEM+AA-P with either 10% or 1% serum for 2 days or 3D in DMEM+AA-P with 10% serum. F) Number of HCT116 cells cultured in 2D in DMEM+AA-P with either 10% or 1% serum for 2 days. G) Intracellular proline in HCT116 cells cultured in 2D at either 37°C or 33°C for 2 days or 3D in DMEM+AA-P. H) Number of HCT116 cells cultured in 2D DMEM+AA-P at either 37°C or 33°C for 2 days. I) Quantification of cell cycle analysis of HCT116 cells grown in DMEM+AA-P in 2D at 37°C and 10% serum or the indicated modifications, or in 3D. J) Western blot showing the expression of the indicated proteins in HCT116 cells cultured in 2D, 3D, at 33°C or in 1% serum in DMEM+AA with or without proline. Vinculin is the loading control. The numbers after each protein refer to the membrane the antibody was incubated with. All metabolites measured using LC-MS. All data presented as mean ± SD, n = 3 technical replicates, except medium where n = 1. Welch’s t-test: * $p < 0.05$, ** $p < 0.01$, *** $p < 0.001$, **** $p < 0.0001$.

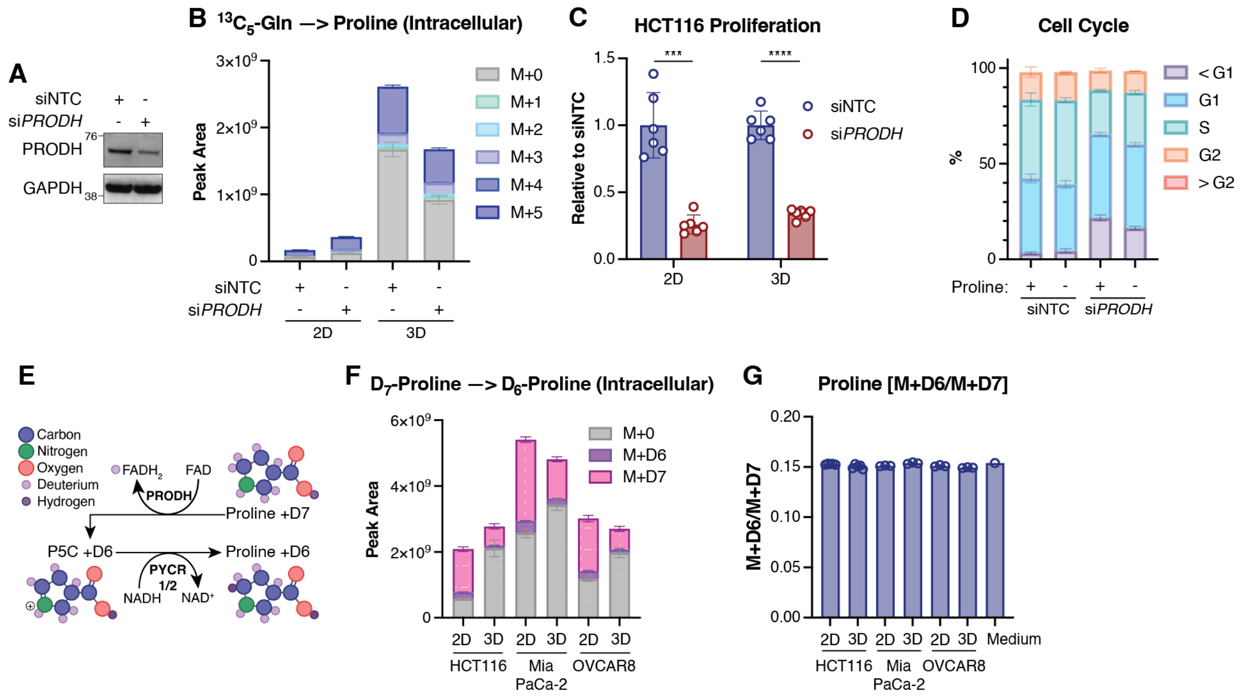

Figure S3: Proline accumulation in 3D does not result from proline recycling.

A) Western blot showing expression of PRODH in HCT116 cells transfected with either a non-targeting control (NTC) siRNA or an siRNA targeting *PRODH*. GAPDH is the loading control. B) Intracellular proline in HCT116 cells in 2D or 3D transfected with either an NTC siRNA or an siRNA targeting *PRODH* in DMEM with $^{13}C_5$-glutamine. N = 5. C) Proliferation measured by cell number on day 5 after seeding of HCT116-iRFP cells in 2D or 3D transfected with either an NTC siRNA or an siRNA targeting *PRODH*. iRFP fluorescence set relative to siNTC. Performed in DMEM+AA+P. N = 6. D) Quantification of cell cycle analysis of HCT116 cells transfected with either an NTC siRNA or an siRNA targeting *PRODH* in DMEM+AA with or without proline. N = 3. E) Schematic showing the conversion of D_7-proline to D_6-proline via PRODH and the PYCR enzymes. F) Intracellular proline in the indicated cell lines cultured in 2D or 3D in DMEM+AA+P with D_7-proline. Measured using LC-MS. G) Ratio of D_6 to D_7-proline from data in F). 'Medium' shows proline level in the medium before it was put onto cells. All metabolites measured using LC-MS. All data presented as mean ± SD, $n \geq 3$, except medium where n = 1. C) Multiple unpaired Welch t-tests corrected for multiple comparisons: *** $p < 0.001$, **** $p < 0.0001$.

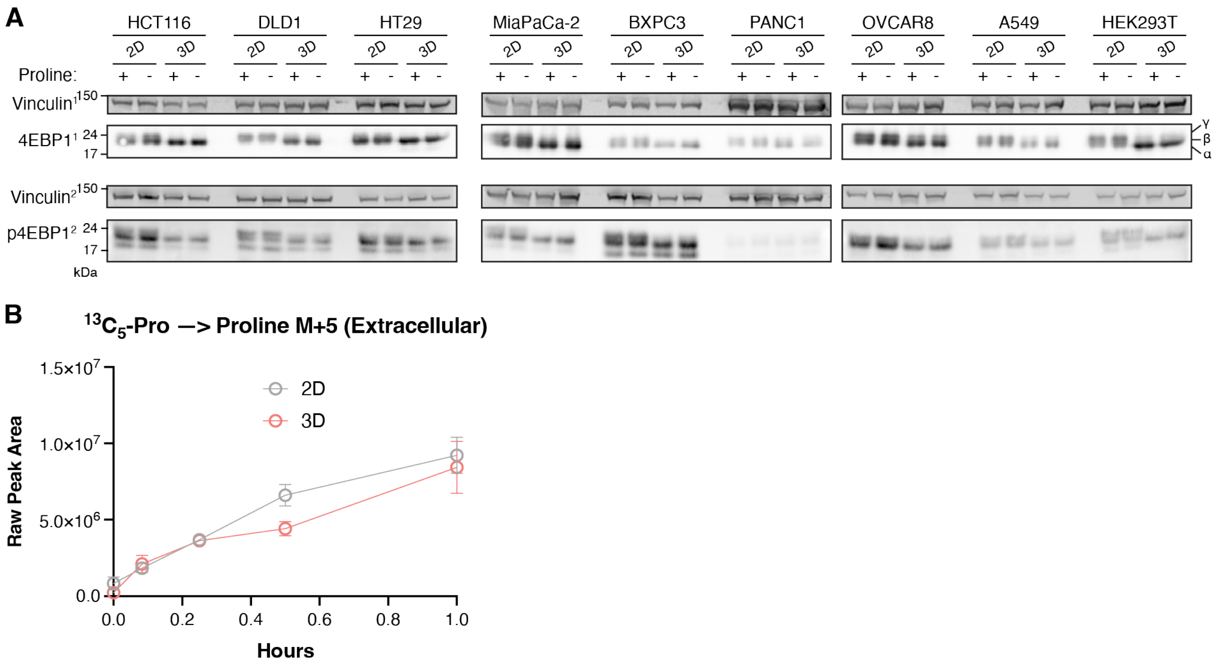

Figure S4: **4EBP1 phosphorylation and excretion of labelled intracellular proline in 2D and 3D.**

A) Western blot showing p4EBP1 and 4EBP1 expression in various cell lines in 2D and 3D in DMEM+AA with or without proline. α indicates unphosphorylated 4EBP1, while β and γ indicate phosphorylated forms of 4EBP1. B) Extracellular M+5 proline in HCT116 P5CS-KO cells cultured in 2D or 3D in DMEM+AA+P with $^{13}C_5$-proline at specific timepoints after medium was changed to DMEM+AA+P with unlabelled proline. Data presented as mean ± SD, n = 3. B) Multiple Welch unpaired t-tests.

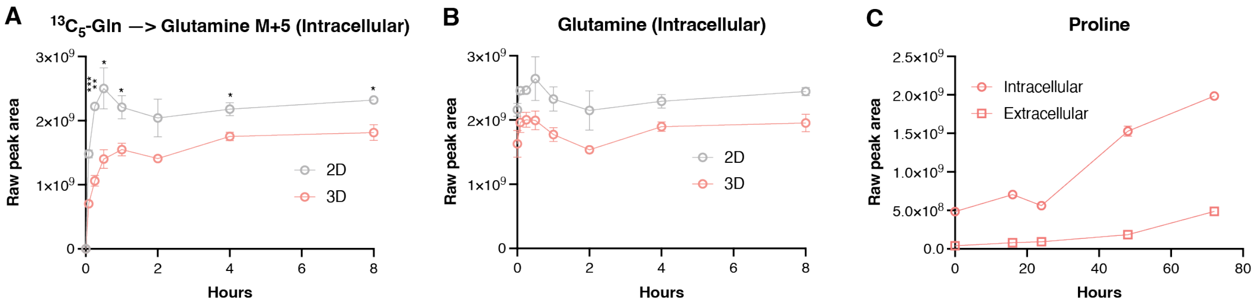

Figure S5: **General amino acid synthesis, but not proline synthesis, is reduced in 3D culture.**

The same experiment shown in Figure 5A over 8 hours A) glutamine M+5, B) total glutamine. C) Intracellular and extracellular proline from metabolite samples taken at defined timepoints from cells seeded in 3D in DMEM+AA-P. N = 3 technical replicates at each timepoint. Data shown as mean ± SD. A) and B) multiple Welch unpaired t-tests, corrected for multiple comparisons: * $p < 0.05$, ** $p < 0.01$, *** $p < 0.001$.
